# Enhanced detection of abnormalities in heart rate variability and dynamics by 7‐day continuous ECG monitoring

**DOI:** 10.1111/anec.12897

**Published:** 2021-09-21

**Authors:** Junichiro Hayano, Emi Yuda

**Affiliations:** ^1^ Heart Beat Science Lab, Co., Ltd. Sendai Japan; ^2^ Nagoya City University Nagoya Japan; ^3^ Center for Data‐driven Science and Artificial Intelligence Tohoku University Sendai Japan

**Keywords:** heart rate dynamics, heart rate variability, Holter ECG, long‐term ECG monitoring, day‐to‐day variation, intraweek variation

## Abstract

**Background:**

The analysis of heart rate variability (HRV) and heart rate (HR) dynamics by Holter ECG has been standardized to 24 hs, but longer‐term continuous ECG monitoring has become available in clinical practice. We investigated the effects of long‐term ECG on the assessment of HRV and HR dynamics.

**Methods:**

Intraweek variations in HRV and HR dynamics were analyzed in 107 outpatients with sinus rhythm. ECG was recorded continuously for 7 days with a flexible, codeless, waterproof sensor attached on the upper chest wall. Data were divided into seven 24‐h segments, and standard time‐ and frequency‐domain HRV and nonlinear HR dynamics indices were computed for each segment.

**Results:**

The intraweek coefficients of variance of HRV and HR dynamics indices ranged from 2.9% to 26.0% and were smaller for frequency‐domain than for time‐domain indices, and for indices reflecting slower HR fluctuations than faster fluctuations. The indices with large variance often showed transient abnormalities from day to day over 7 days, reducing the positive predictive accuracy of the 24‐h ECG for detecting persistent abnormalities over 7 days. Conversely, 7‐day ECG provided 2.3‐ to 6.5‐fold increase in sensitivity to detect persistent plus transient abnormalities compared with 24‐h ECG. It detected an average of 1.74 to 2.91 times as many abnormal indices as 24‐h ECG.

**Conclusions:**

Long‐term ECG monitoring increases the accuracy and sensitivity of detecting persistent and transient abnormalities in HRV and HR dynamics and allows discrimination between the two types of abnormalities. Whether this discrimination improves risk stratification deserves further studies.

## 1 INTRODUCTION

The standard recording time for Holter ECGs has long been 24 h, but recently, continuous ECG monitoring for longer than a week has become available in clinical practice. Several studies have indicated inadequate detection rates of 24‐h ECG monitoring for infrequent but clinically significant arrhythmic events, such as paroxysmal atrial fibrillation, and they reported that it could be improved by extending the monitoring time (Brachmann et al., 2016; Hariri et al., 2016; Liao et al., 2007). In a study comparing arrhythmia detection with 14‐day continuous ECG and 24‐h Holter monitoring in 32 patients, Chua et al. (2020) reported that the former detected 202 episodes of paroxysmal atrial fibrillation/flutter in 6 patients, whereas the latter detected only 1 episode of paroxysmal atrial fibrillation in 1 patient. Extended ECG monitoring for multiple days may uncover new ECG features that were not observed in conventional 24‐h Holter ECG.

The duration of heart rate variability (HRV) analysis has also been standardized to 24 h. Analysis of HRV and nonlinear heart rate (HR) dynamics of Holter ECG has been mainly used to predict increased risk of poor prognosis in cardiac diseases, and the major predictive indices of HRV and HR dynamics including standard deviation of normal‐to‐normal R‐R interval (SDNN) (Camm et al., 1996; Kleiger et al., 1987), deceleration capacity (DC) (Bauer et al., 2006; Kantelhardt et al., 2007), scaling exponent α_1_ by detrended fluctuation analysis (DFA) (Huikuri et al., 2000; Iyengar et al., 1996; Peng et al., 1995), and very‐low‐frequency (VLF) power (Bigger et al., 1992) are all calculated in units of 24 h. Earlier studies have reported stability of HRV indices over time by showing good reproducibility between two measurements made on different days (Kleiger et al., 1991; Van Hoogenhuyze et al., 1991), but the level of day‐to‐day variations during long‐term monitoring has not been studied. In the present study, we investigated the intraweek variation in the indices of HRV and HR dynamics, particularly of the appearance of their abnormalities predicting poor prognosis, during a week.

## 2 METHODS

### 2.1 Continuous ECG monitoring sensor

We used a patch ECG sensor (Heartnote^®^, JSR Corporation) for long‐term monitoring. The sensor was flexible, codeless, electrode‐integrated, waterproof, 30 × 100 mm, 5 mm thick, and 12 g in weight. The sensor was attached on the upper chest wall with adhesive tape and continuously recorded ECG at 256 Hz and triaxial acceleration at 32 Hz for 7 days and stored in it. The sensors were loaned to clinics, attached to patients, collected after measurements, and returned to JSR Corporation, where stored data were extracted, all QRS complexes in ECG were detected, noise and arrhythmia types were annotated, and annotated R‐R interval time series data were generated. These processes were performed on a long‐term Holter ECG analysis viewer (NEY‐HEA3000, Nexis Co., Ltd.) using a Holter analyzer program (JMDN 36827012, Nexis Co., Ltd.), which has been approved by the Japanese Ministry of Health, Labour, and Welfare (Medical device approval number 228AGBZX00099000). To annotate the cardiac rhythms, the analyzer program classified QRS complexes by the standard cycle length criteria for supraventricular ectopic heartbeats, grouped them by QRS morphology, and labeled the groups according to the type of arrhythmia. The results of the automated analysis were reviewed and edited by skilled technicians, and the morphological classification table was provided to medical doctors for confirmation. The long‐term R‐R interval time series thus generated were provided for this study.

### 2.2 Study design

We studied data in consecutive 158 outpatients (67 males, 71 females, and 20 of unknown gender; age ± SD, 64 ± 16 years) who underwent 7‐day continuous ECG monitoring for the screening or evaluation of arrhythmias between August 2020 and January 2021 in Japan. The inclusion criteria were (1) aged 20 years or older, (2) provision of written informed consent for the use of the anonymized data in this study, (3) sinus rhythm in 12‐lead ECG at the entry, and (4) willingness to comply with up to 7 days of continuous ECG monitoring.

The written informed consent was obtained from each subject by JSR Corporation (Japan). The protocol was approved by the Ethics Review Committee of the Nagoya City University Graduate School of Medical Sciences, Nagoya, Japan (No. 60–18–0211).

### 2.3 Data selection

The continuous R‐R interval time series for a week was divided into 24‐h intervals from the beginning of the monitoring. Only those 24‐h data segments in which the total duration of sinus rhythm was ≥19.2 (24 × 0.8) h were used as valid data segments. Then, only those patients who had six or more valid 24‐h data segments during a week were included for the final analysis.

### 2.4 Computations of HRV and HR dynamics indices

For each valid 24‐h data segment, we computed the time‐domain and frequency‐domain indices of HRV and nonlinear indices of HR dynamics that are used for predicting increased cardiovascular mortality risk and for assessing autonomic functions. They were computed by the methods according to the recommended standard (Camm et al., 1996) and to the earlier studies (Iyengar et al., 1996; Kantelhardt et al., 2007; Peng et al., 1995). Briefly, from the ECG data, the time series of N‐N intervals, $\left\{NN_{i}\right\}=\left\{tN_{i}‐tN_{i‐1}\right\}$, where *tNi* represents the time of occurrence of the *i*‐th normal sinus beat were derived. For the time‐domain HRV indices, mean normal‐to‐normal interval (MNN) and SDNN were computed as 24‐h mean and standard deviation of *NNi*, respectively. The standard deviation of average N‐N interval (SDANN) was calculated as the standard deviation of the averages of N‐N interval of nonoverlapping 5‐min segments over 24 h, and the root mean square of successive N‐N interval difference (rMSSD) was obtained as the square root of average squared difference between all successive N‐N intervals during 24 h. DC was computed by the phase rectified signal averaging of the 24‐h N‐N interval time series (Kantelhardt et al., 2007). For a frequency‐domain index, we computed the power of ultra‐low, very‐low, low, and high frequency (ULF, VLF, LF, and HF, respectively) components. For this purpose, 24‐h {*NNi*} time series were interpolated by a horizontal step function, resampled at 2 Hz, filtered with a Hanning window, and converted into the frequency domain by a fast Fourier transform (FFT). The power spectral density was integrated for the power within the ULF ( < 0.0033 Hz), VLF (0.0033–0.04 Hz), LF (0.04–0.15 Hz), and HF (0.15–0.4 Hz) bands. The power of these components was transformed into natural logarithmic values to normalize the distribution. LF/HF was calculated as the ratio of the absolute values of LF power and HF power, and the spectral exponent β was calculated as the slope of the log‐log plot of the 24‐h power spectrum (Bigger et al., 1996). For the nonlinear indices, we calculated the fractal correlation properties of HR dynamics using the DFA method and measured the short‐term (4 to 11 beat) and long‐term (>11 beats) scaling exponents (α_1_ and α_2_, respectively) (Iyengar et al., 1996; Peng et al., 1995).

### 2.5 Data analysis

To assess the day‐to‐day variation in the indices of HRV and HR dynamics, we calculated the intraweek coefficient of variance. First, the indices of HRV and HR dynamics were calculated for each 24‐h segment during the monitoring week (7 days). Next, for each index, the average and standard deviation (SD) of the 24‐h values during the 7 days were calculated in each patient. Then, the intraweek coefficient of variance was calculated in each patient using the following formula.

$$\text{Coefficient}\;\text{of}\;\text{variance}\;\left(\%\right)=100\times \frac{\text{SD}\;\text{of}\;24\;\text{hour}\;\text{index}\;\text{during}\;\text{week}}{\text{Mean}\;\text{of}\;24\;\text{hour}\;\text{index}\;\text{during}\;\text{week}}$$

We examined the day‐to‐day variation in the occurrence of abnormal values of HRV and HR dynamics during the monitoring week. This analysis was performed only for the HRV and HR dynamics indices known as the major predictors of increased risk for mortality after acute myocardial infarction (AMI), which included SDNN, DC, DFA α_1_, ULF, VLF, LF, and LF/HF (Bauer et al., 2006; Hayano et al., 2021; Huikuri et al., 2000; Yuda et al., 2020). The abnormal values of HRV and HR dynamics were defined for each index using values reported in previous studies as cutoffs for stratifying the level of mortality risk (Bauer et al., 2006; Huikuri et al., 2000; La Rovere et al., 2003).

To compare the ability of 7‐day ECG to detect abnormalities in HRV and HR dynamics with 24‐h ECG, the following two types of abnormalities were defined for each index of HRV and HR dynamics.

1. Index is abnormal in 7‐day average (type 1 abnormality)
2. Index is abnormal at least on one day during 7‐day monitoring (type 2 abnormality)

For type 1 abnormality, the sensitivity, specificity, and positive and negative predictive accuracies were calculated for each index by analyzing the confusion matrix between the presence of type 1 abnormality and the detection of abnormal value by 24‐h segmented ECG. For type 2 abnormality, the improvement of the detection of abnormality by 7‐day continuous ECG monitoring was estimated by the expected sensitivity ratio, S_7‐day_/S_24‐h_, where S_7‐day_ is the sensitivity of 7‐day continuous ECG monitoring to detect type 2 abnormality and S_24‐h_ is the sensitivity of 24‐h ECG to detect the abnormal value of the index. Since type 2 abnormality was defined by 7‐day data, S_7‐day_ is always 100% by that definition in this study. S_24‐h_ was estimated as the probability that the abnormal values would be detected by 24‐h ECG monitoring on one day of the 7 days (E_24‐h_). Then, the sensitivity ratio was estimated using the following equation.

$$S_{7‐\text{day}}/S_{24‐\text{hour}}\approx 1/E_{24‐\text{hour}}$$

Since the specificity to detect type 2 abnormality by 24‐h ECG is always 100% by the definition of type 2 abnormality, only expected sensitivity was evaluated for type 2 abnormality.

The improvement of the detection rate of the number of indices with abnormal values (abnormal indices) by 7‐day continuous ECG monitoring was estimated by the expected detection ratio of abnormal indices. To calculate this ratio, patients were grouped by the number of abnormal indices detected during 7‐day ECG monitoring (C_7‐day_), using only seven abnormal indices, including SDNN < 70 ms, DC < 4.5 ms, DFA α1 < 0.75, ULF < 8.1 ln (ms^2^), VLF < 5.75 ln(ms^2^), LF < 5.5 ln(ms^2^), and LF/HF < 0.43. In each patient, the average and maximum number of abnormal indices detected by 24‐h ECG segments (C_24‐h_) during the 7 days were calculated. Then, the average and minimum expected detection ratio of abnormal indices of 7‐day ECG monitoring (R_7‐day_) were computed as (C_7‐day_/average C_24‐h_) and (C_7‐day_/maximum C_24‐h_), respectively, in each patient.

### 2.6 Statistical analysis

SAS program package version 9.4 (SAS Institute) was used for statistical analyses. The sex differences in mean values and frequency were evaluated by *t*‐test and chi‐squared test, respectively. Repeated measures analysis of variance (ANOVA) by GLM procedure was used to compare the coefficient of variance among different HRV and HR dynamics indices. Post hoc multiple comparisons between two coefficients were performed with generating contrasts between adjacent levels of the coefficient. Considering the non‐Gaussian distribution of variables, the correlations between two variables were evaluated by Spearman's coefficient of rank correlation. The confusion matrices were generated by FREQ procedure. Statistical significance was determined with an α < .05 with Bonferroni adjustment.

## 3 RESULTS

### 3.1 Characteristics of selected data

Of the 158 patients who met the inclusion criteria, 51 patients were excluded because they did not have at least six segments of a 24‐h ECG in sinus rhythm over 19.2 (24 × 0.8) h. The reasons for not obtaining the required number of valid 24‐h ECG segments were excessive noises in 39 (24.7%) patients, long episode(s) of paroxysmal atrial fibrillation in 5 (3.2%) patients, and frequent arrhythmias in 7 (4.4%) patients. The remaining 107 patients were included in the final analysis (Table 1).

**TABLE 1:** Patients' characteristics

	Male	Female	Total
*N* (%)	43 (40%)	52 (49%)	107 (100%)
Age, year			
Mean ± SD	64 ± 15	64 ± 16	64 ± 15
Median (IQR)	65 (55–75)	65 (55–75)	65 (55–75)
Monitoring period, day			
Mean ± SD	6.7 ± 0.5	6.6 ± 0.5	6.7 ± 0.5
Median (IQR)	7 (6–7)	7 (6–7)	7 (6–7)
≥7 days, N (%)	30 (70%)	33 (63%)	72 (67%)

### 3.2 Intraweek variations in HRV and HR dynamics indices

**FIGURE 1:**
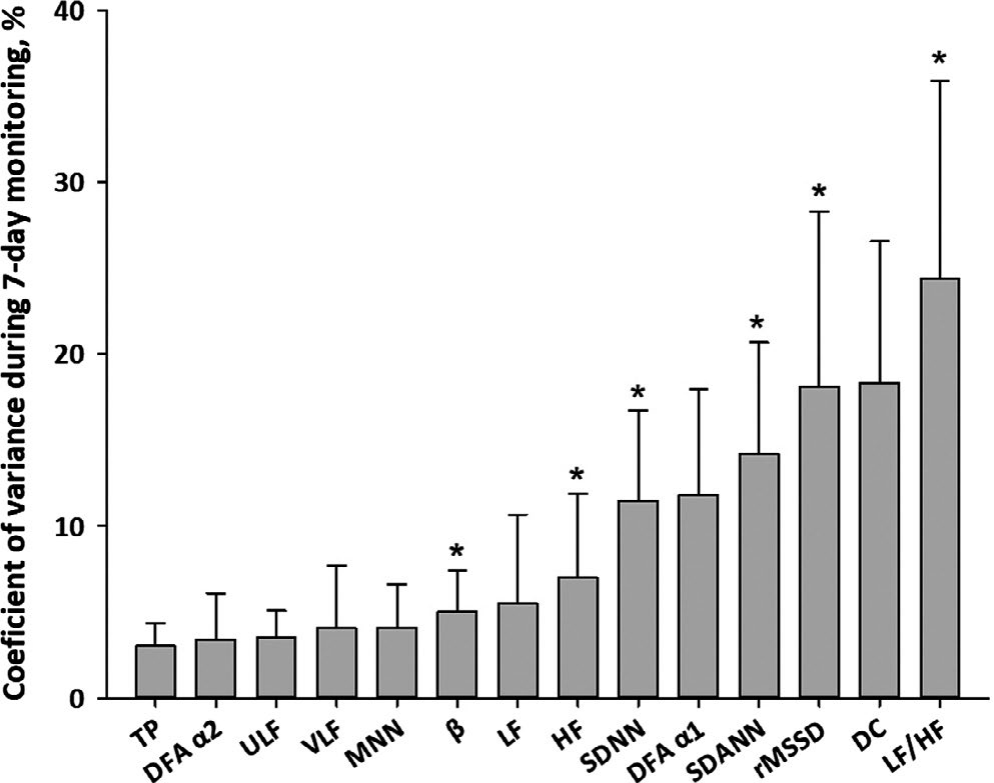
Coefficients of variance of HRV indices during 7‐day ECG monitoring (total sample, *N* = 107). *Significantly different from adjacent values on the left (*p* < .005, Bonferroni adjustment for 13 multiple comparisons). Error bars indicate SD

Table 2 shows intraweek average and coefficient of variance of the indices of HRV and HR dynamics by gender. The intraweek coefficient of variance differed between indices in both genders, ranging from 2.9% to 26.0%, although no significant sex difference was observed. Figure 1 shows the coefficient of variance of each index in total sample, sorted by the magnitude of the values. The values differed among indices (*p* < .0001), with frequency‐domain indices (TP, ULF, VLF, LF, and HF) showing less intraweek variation than time‐domain indices (SDNN, SDANN, and rMSSD), and indices reflecting slow fluctuations (TP, DFA α_2_, ULF, VLF, and MNN) showing less variation than indices reflecting fast fluctuations (LF, HF, SDNN, DFA α_1_, rMSSD, DC, and LF/HF).

**TABLE 2:** Intraweek variation in heart rate variability and heart rate dynamics

	Average			Coefficient of variance, %		
	Male	Female	p*	Male	Female	p*
MNN, ms	820 ± 95	839 ± 108	.3	4.7 ± 2.9	3.7 ± 2.2	.08
SDNN, ms	132 ± 38	127 ± 26	.4	11.5 ± 5.1	11.9 ± 5.6	.4
SDNAN, ms	118 ± 39	113 ± 29	.5	13.3 ± 6.3	14.9 ± 6.9	.2
rMSSD, ms	54 ± 24	58 ± 23	.4	19.0 ± 9.7	17.5 ± 11	.4
DC, ms	7.7 ± 2.2	7.2 ± 1.6	.2	18.9 ± 10.1	18.1 ± 6.9	.6
DFA α_1_	0.83 ± 0.21	0.72 ± 0.20	.01	12.9 ± 7.5	11.0 ± 4.9	.1
DFA α_2_	1.15 ± 0.05	1.16 ± 0.05	.09	3.9 ± 3.6	3.1 ± 1.8	.1
β	1.29 ± 0.09	1.28 ± 0.09	.8	5.3 ± 2.8	4.7 ± 2.2	.2
TP, ln(ms^2^)	9.7 ± 0.5	9.7 ± 0.6	.9	3.1 ± 1.5	2.9 ± 1.3	.4
ULF, ln(ms^2^)	9.5 ± 0.6	9.5 ± 0.7	.9	3.7 ± 1.5	3.4 ± 1.6	.4
VLF, ln(ms^2^)	7.1 ± 0.7	6.9 ± 0.6	.3	4.6 ± 4.8	3.9 ± 2.8	.3
LF, ln(ms^2^)	5.8 ± 0.9	5.7 ± 0.7	.3	6.2 ± 6.4	5.3 ± 4.3	.4
HF, ln(ms^2^)	5.7 ± 0.8	5.8 ± 0.7	.3	8.0 ± 6.1	6.4 ± 3.9	.1
LF/HF	1.3 ± 0.7	1.0 ± 0.6	.01	26.0 ± 11.8	23.4 ± 10.7	.2

Note

Values are mean ± SD.

*Significance of gender difference.

### 3.3 Positive rate of type 1 and type 2 abnormality in HRV and HR dynamics of 7‐day ECG

Table 3 shows the positive rate of type 1 and type 2 abnormalities of 7‐day HRV and HR dynamics. Positive rates of both types of abnormality ranged widely, depending on the index, the cutoff value of the index, and gender. Although type 2 abnormality showed a greater positive rate than type 1 abnormality for all indices, the ratio of positive rate (type 2/type 1) was larger for indices with a large coefficient of variance (SDNN < 70 ms and DC < 4.5 ms), indicating that these indices showed frequent daily transient appearance of abnormal value.

**TABLE 3:** Positive rate of type 1 and type 2 abnormalities of 7‐day HRV and HR dynamics

Definition of abnormality	Type 1 abnormality			Type 2 abnormality		
	Male %	Female %	Total %	Male %	Female %	Total %
SDNN < 65 ms	0.00	0.00	0.00	2.3	3.9	2.8
SDNN < 70ms	2.08*	0.00	0.84	4.7	9.6	6.5
DC ≤ 2.5 ms	0.00	0.00	0.00	2.4	1.9	1.9
DC < 4.5 ms	4.18*	1.74	2.52	28.6	36.5	31.1
α_1_ < 0.75	44.4	67.5†	52.5	60.5	76.9	71.0
ULF < 8.1 ln(ms^2^)	0.00	0.00	0.00	11.6	7.7	8.4
VLF < 5.75 ln(ms^2^)	4.51*	1.74	2.66	18.6	9.6	12.2
LF < 5.5 ln(ms^2^)	34.4	34.8	32.3	53.5	61.5	55.1
LF/HF < 0.43	0.00	11.3†	5.46	9.3	30.8†	20.6

Note

Type 1 abnormality, index was abnormal in 7‐day average. Type 2 abnormality, index was abnormal at least on one day during 7‐day monitoring.

*Significantly greater in male; †Significantly greater in female.

### 3.4 Sensitivity and specificity to detect type 1 abnormality by 24‐h ECG

Table 4 shows the sensitivity and specificity of 24‐h segmented ECG analysis for detection of type 1 abnormality of 7‐day HRV and HR dynamics. For all evaluable indices, 24‐h ECG showed high specificity and negative predictive accuracy (>89.8% and >86.3% for all samples, respectively), whereas, the sensitivity and positive predictive accuracy varied greatly among the indices with the latter being particularly low for DC < 4.5 ms (12.3%), SDNN < 70 ms (23.8%), and VLF < 5.75 ln(ms^2^) (41.9%), indicating that 24‐h ECG had a high false positive rate for type 1 abnormality of these indices.

**TABLE 4:** Sensitivity and specificity of 24‐h ECG for detection of type 1 abnormality of 7‐day HRV and HR dynamics

Definition of abnormality	Sensitivity, %			Specificity, %			Positive predictive accuracy, %			Negative predictive accuracy, %		
	Male	Female	Total	Male	Female	Total	Male	Female	Total	Male	Female	Total
SDNN < 65 ms	–	–	–	–	–	–	–	–	–	–	–	–
SDNN < 70ms	50.0	–	50.0	98.9	–	98.6	50.0	–	23.8	98.9	–	99.6
DC ≤ 2.5 ms		–	–	–	–	–	–	–	–	–	–	–
DC < 4.5 ms	41.7	50.0	44.4	91.6	91.5	91.8	17.9	9.38	12.3	97.3	99.0	98.5
α_1_ < 0.75	82.8	88.4	86.9	93.8	92.0	91.2	91.4	95.8	91.6	87.2	79.2	86.3
ULF < 8.1 ln(ms^2^)	–	–	–	–	–	–	–	–	–	–	–	–
VLF < 5.75 ln(ms^2^)	61.5	83.3	68.4	96.4	97.6	97.4	44.4	38.5	41.9	98.2	99.7	99.1
LF < 5.5 ln(ms^2^)	83.8	96.7	85.8	91.0	86.7	89.8	83.0	77.6	80.3	91.5	92.4	92.9
LF/HF < 0.43	–	69.2	69.2	–	95.4	96.6	–	65.9	54.0	–	96.1	98.2

### 3.5 Estimated sensitivity ratio for type 2 abnormality by 7‐day ECG

**FIGURE 2:**
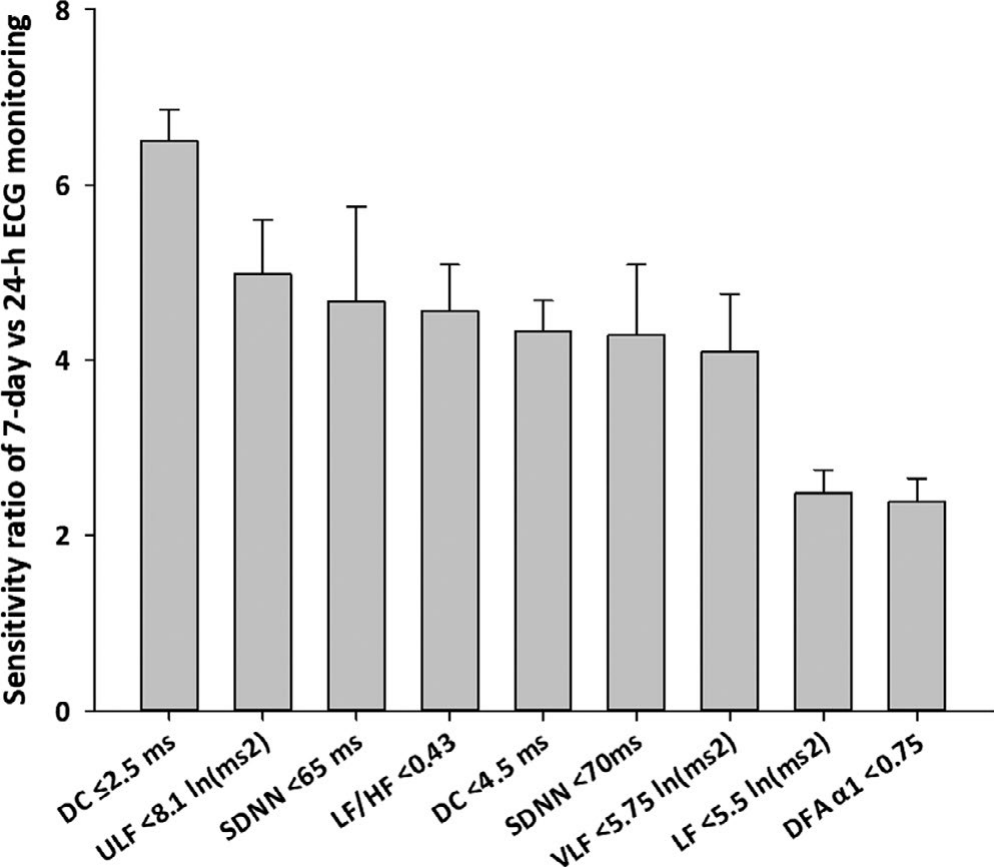
Expected sensitivity ratio of 7‐day ECG monitoring to 24‐h ECG monitoring for detecting type 2 abnormalities in HRV and HR dynamics. Data are means and standard error of the means (error bars) over total sample (*N* = 107)

Table 5 shows estimated sensitivity ratio for detection of type 2 abnormality by 7‐day ECG monitoring compared to 24‐h ECG. The ratio varied depending on the index and cutoff value, with SDNN < 65 ms and DC < 4.5 ms showing a large ratio in females and for LF/HF < 0.43 in males. Figure 2 shows the estimated sensitivity ratio of each abnormal index in total sample, sorted by the magnitude of the ratios. The ratio differed for each index, and was negatively correlated with the positive rate of type 2 abnormality (Table 3; Spearman's coefficient of rank correlation = −0.78, *p* = 0.01), but it was not associated significantly with the intraweek coefficient of variance (Figure 1; Spearman's coefficient of rank correlation =0.52, *p* = .1).

**TABLE 5:** Expected sensitivity ratio for detection of type 2 abnormality by 7‐day ECG compared with 24‐h ECG

Definition of abnormality	Expected sensitivity ratio (1/E_24‐h_)		
	Male Mean ± SEM	Female Mean ± SEM	p*
SDNN < 65 ms	2.00 ± 0.00	6.00 ± 0.00	–
SDNN < 70ms	2.00 ± 0.00	5.20 ± 0.82	.02
DC ≤ 2.5 ms	7.00 ± 0.00	6.00 ± 0.00	–
DC < 4.5 ms	3.46 ± 0.39	5.07 ± 0.48	.02
α_1_ < 0.75	2.45 ± 0.43	1.66 ± 0.25	.09
ULF < 8.1 ln(ms^2^)	6.00 ± 0.69	3.71 ± 0.69	.08
VLF < 5.75 ln(ms^2^)	4.56 ± 0.88	3.34 ± 0.89	.4
LF < 5.5 ln(ms^2^)	2.24 ± 0.37	2.47 ± 0.36	.6
LF/HF < 0.43	7.00 ± 0.00	4.16 ± 0.64	.0006

Abbreviations: 1/E_24‐h_, expected sensitivity ratio of 7‐day ECG monitoring to 24‐h monitoring for detecting type 2 abnormality; E_24‐h_, Probability of 24‐h ECG to detect type 2 abnormality; SEM, standard error of mean.

*Significance of gender difference.

### 3.6 Number of abnormal indices of HRV and HR dynamics detected by 7‐day ECG

Table 6 shows the number of abnormal HRV and HR dynamics indices detected by 7‐day ECG and 24‐h ECG segments (C_7‐day_ and C_24‐h_). The maximum C_24‐h_ indicates the degree of the overlapping occurrence of multiple abnormal indices on the same days (C_24‐h_ =1 indicates no overlap and C_24‐h_ = C_7‐day_ indicates complete overlap). When multiple abnormal indices appeared during 7 days (C_7‐day_ >1), the actual maximum C_24‐h_ was intermediate, indicating that multiple abnormal indices partially overlapped on the same days during a week. Consequently, as indicated by R_7‐day_, 7‐day ECG monitoring was expected to detect 1.74 to 2.91 times on average and 1.16 to 1.39 times at minimum as many abnormal values as 24‐h ECG monitoring.

**TABLE 6:** Number of abnormal HRV and HR dynamics indices detected during 7‐day monitoring and that expected in 24‐h monitoring

Number of detected abnormal indices				Expected detection ratio for 7‐day monitoring		
C_7‐day_	*N* of patients	C_24‐h_		Average R_7‐day_	Minimum R_7‐day_	
		Average	Maximum	C_7‐day_/average C_24‐h_	C_7‐day_/maximum C_24‐h_	
1	18	0.62 ± 0.08	1.00 ± 0.00	2.91 ± 0.60		1.00 ± 0.00
2	25	1.02 ± 0.08	1.84 ± 0.07	2.51 ± 0.33		1.16 ± 0.07
3	19	1.47 ± 0.12	2.53 ± 0.12	2.39 ± 0.25		1.24 ± 0.06
4	8	2.23 ± 0.08	3.13 ± 0.13	1.81 ± 0.06		1.29 ± 0.04
5	9	2.68 ± 0.25	3.89 ± 0.35	2.09 ± 0.31		1.39 ± 0.16
6	3	3.57 ± 0.50	5.00 ± 0.58	1.74 ± 0.24		1.23 ± 0.15

Note

The number of abnormal indices among SDNN < 70 ms, DC < 4.5 ms, DFA α_1_ < 0.75, ULF < 8.1 ln(ms^2^), VLF < 5.75 ln(ms^2^), LF < 5.5 ln(ms^2^), LF/HF < 0.43. Data are mean ± SEM.

Abbreviations: average R_7‐day_, C_7‐day_/average C_24‐h_; C_24‐h_, the number of abnormal indices detected by 24‐h ECG monitoring; C_7‐day_, the number of abnormal indices detected during 7‐day ECG monitoring; minimum R_7‐day_, C_7‐day_/maximum C_24‐h_.

## 4 DISCUSSION

To our knowledge, this is the first study to examine the impacts of long‐term continuous ECG monitoring on the evaluation of HRV and HR dynamics. In this study, we analyzed intraweek variations in HRV and HR dynamics indices during 7‐day continuous ECG monitoring in 107 outpatients who maintained sinus rhythm during the monitoring. The results showed that HRV and HR dynamics indices, calculated on a 24‐h basis, showed intraweek coefficients of variance ranging from 2.9% to 26.0%. The performance of 7‐day ECG monitoring in detecting abnormalities in HRV and HR dynamics relative to 24‐h ECG monitoring was evaluated for type 1 (persistent) and type 2 (persistent plus transient) abnormalities. The indices with large variance including DC, SDNN, and VLF often showed transient abnormalities from day to day over 7 days, reducing the positive predictive accuracy of the 24‐h ECG monitoring for detecting type 1 abnormality of these indices. In other words, 7‐day ECG monitoring can distinguish between persistent and transient abnormalities that 24‐h ECG monitoring cannot. For the detection of type 2 abnormalities, 7‐day ECG monitoring was estimated to provide 2.3‐ to 6.5‐fold increase in sensitivity compared to 24‐h ECG monitoring. Also, as to the number of abnormal indices, 7‐day ECG monitoring was estimated to detect 1.74 to 2.91 times on average and 1.16 to 1.39 times at minimum of multiple abnormal indices compared with 24‐h ECG monitoring.

Earlier studies reported stability over time of HRV indices (Kleiger et al., 1991; Van Hoogenhuyze et al., 1991). In a study comparing 24‐h HRV indices measured at baseline and on placebo medication (3–65 days apart) in 14 normal subjects aged 22 to 55 years, Kleiger et al. (1991) reported intraclass correlation coefficients and standard errors of mean (SEM) between two measures of time‐domain and frequency‐domain HRV indices. The variances of measures estimated from $\sqrt{2}\times$ SEM of the study were 5.7, 15.4, 13.9, 21.8, 2.9, 3.0, and 5.9% of means for MNN, SDNN, SDANN, rMSSD, TP, LF, and HF, respectively. These values are comparable to the intraweek coefficients of variance observed for corresponding indices in the present study and show a similar negative relationship to the cycle length of fluctuation that the indices reflect. Van Hoogenhuyze et al. (1991) examined the reproducibility of time‐domain indices of 24‐h HRV on two successive days in 33 normal subjects aged 24–57 years and 22 outpatients aged 45–71 years with coronary artery disease and prior AMI. They reported that MNN, SDNN, and SDANN showed mean day‐to‐day differences of 4.1, 7.8, and 12.3%, respectively, in normal subjects and 2.8, 8.5, and 13.8%, respectively, in outpatients. These values are also comparable to the intraweek variations in the present study.

The difference between these earlier studies (Kleiger et al., 1991; Van Hoogenhuyze et al., 1991) and the present study lies not only in the duration of the continuous monitoring, but also in the possible effects of monitoring on subjects’ behaviors. In separated 24‐h monitoring, the subject has to visit the laboratory twice, before and at the end of the monitoring, for the installation and removal of electrodes, which restricts subjects’ daily schedule and activities accordingly, whereas in 7‐day continuous monitoring, the subjects free to move around except for the first and last days of monitoring. Nevertheless, the levels of day‐to‐day variation in the HRV indices were similar between them. This suggests that the changes in the subject's activity due to monitoring itself do not have a large effect on the day‐to‐day variation in the HRV indices.

On the other hand, we observed that indices of HRV and HR dynamics that reflect fast fluctuations (LF, HF, SDNN, DFA α1, rMSSD, DC, and LF/HF) showed greater day‐to‐day variations than indices that reflect slow fluctuations (TP, DFA α2, ULF, VLF, and MNN), which is also consistent with earlier observations (Kleiger et al., 1991). Although the mechanisms of this phenomenon cannot be determined from this observational study, the cycle‐length‐dependent effects of daily physical activities on HR fluctuations may be involved. HR fluctuation at frequency below the LF band consists mostly of nonlinear fluctuation with scale‐independent fractal properties (Saul et al., 1988). Nunes Amaral et al. (2001) reported that most of the nonlinear component of HR fluctuation remained even under a constant routine protocol with minimal changes in physical activities and posture, suggesting one of the mechanisms by which slow HR fluctuations is less sensitive to changes in daily activity. HR fluctuations at frequency of LF band and above are known to be affected by body posture (Pomeranz et al., 1985), physical activity (Yamamoto et al., 1991), and emotional states (Zhu et al., 2019). Therefore, day‐to‐day differences in physical activities and emotional states may have stronger impact on the indices reflecting fast HR fluctuations than on the indices reflecting slow HR fluctuations.

Additionally, time‐domain HRV indices such as SDNN, SDANN, and rMSSD showed greater intraweek coefficients of variance than frequency‐domain indices that reflect HRV in quasi‐corresponding frequency bands, that is, TP, ULF, and HF, respectively. This may be resulted at least partly from the effect of interpolation of the removed non‐NN intervals, which is done only for the analysis of frequency‐domain indices. The correlation coefficients between SDNN and TP, between SDANN and ULF, and between rMSSD and HF were 0.81, 0.79, and 0.67, respectively, for the whole sample, but 0.83, 0.82, and 0.76, respectively, for 24‐h ECG data that consisted of >95% sinus beats, and 0.73, 0.68, and 0.60, respectively, for 24‐h ECG data that included >5% nonsinus beats or noise (Data not shown). For data containing many non‐NN intervals, the quasi‐correspondence between time‐domain and frequency‐domain indices may be lost as the result of increased fractions of interpolated intervals.

From a clinical standpoint, the most important finding of this study is that 7‐day ECG monitoring actually has an advantage over 24‐h ECG monitoring in terms of positive predictive accuracy for detecting type 1 abnormalities in HRV and HR dynamics, and sensitivity for detecting type 2 abnormalities. While 24‐h segmented ECG analysis had high specificity and negative predictive accuracy (>89.8% and >86.3%, respectively), the sensitivity and positive predictive accuracy was low particularly for DC < 4.5 ms (12.3%), SDNN < 70 ms (23.8%), and VLF < 5.75 ln(ms^2^) (41.9%). We also found that 7‐day ECG monitoring provided 2.3‐ to 6.5‐fold increase in sensitivity to detect type 2 abnormalities compared to 24‐h ECG segment. While these are expected results of extended monitoring period, these facts cannot be ignored clinically given that these indices may be used to stratify mortality risk. If persistent abnormalities of the indices have greater predictive power than transient abnormalities, then identification of the two types abnormalities by 7‐day ECG monitoring may improve risk stratification. Conversely, if transient abnormalities also have predictive power, then more than a fourfold improvement in sensitivity to detect them by 7‐day ECG would contribute greatly risk stratification.

We also observed that7‐day ECG monitoring detected an average of 1.74 to 2.91 times as many abnormal values as 24‐h ECG segments. In this study, we analyzed the degree of overlapping occurrence of multiple abnormal indices on the same days (Table 6). If multiple (7 at maximum in this study) abnormal indices occurred on completely different days within 7 days and did not overlap each other, the maximum C_24‐h_ should be 1.0 regardless of the number of abnormal indices. The actual observed maximum C_24‐h_ were >1.0, indicating that multiple abnormalities overlapped to some extent on the same days. On the other hand, if all multiple abnormalities occurred on the same day, the maximum C_24‐h_ should be equal to C_7‐day_. The actual maximum C_24‐h_ were < C_7‐day_, indicating partial overlap of multiple abnormal indices on the same days during the week. Consequently, it was estimated that 7‐day ECG monitoring could detect about twice as many abnormal as 24‐h ECG, as indicated by R_7‐day_. In a recent study of predictive value of HRV in 138 post‐AMI patients with preserved (≥35%) left ventricular ejection fraction (LVEF), Liu et al. (Liu et al., 2020) reported that decreased SDNN, VLF, and DC were independently associated with increased risk of sudden arrhythmic death and that combination of SDNN, VLF, and DC may help identify a high‐risk patient group. In an earlier study in 687 post‐AMI patients of ENRICHD cohort (Berkman et al., 2003), we also examined the predictive value of combinations of abnormal HRV and HR dynamics indices and found that the combinations of best predictive power of mortality risk differs between patients with reduced (≤35%) and preserved (>35%) LVEF (Hayano et al., 2021). These facts suggest that accurate detection of the number and combination of abnormal indices is important for risk stratification according to the patient's pathophysiologic state after AMI. In this context, the increased detection of abnormal HRV and HR dynamics by long‐term ECG monitoring may have important clinical implications. Future prospective studies in this regard are urgently needed and may facilitate the widespread use of long‐term ECG monitoring.

This study has limitations. First, the study population consisted of consecutive outpatients who underwent 7 days of continuous ECG monitoring for screening or evaluation of arrhythmias, with at least 6 days of 24‐h ECG segments in sinus rhythm for at least 19.2 hs, but no other information, including underlying medical conditions, was available. The generalizability of the present findings needs to be examined by future studies. Second, the abnormalities of HRV and HR dynamics indices were defined using reported cutoffs to predict mortality risk in patients after acute myocardial infarction. Therefore, the prognostic value of these indices in the present population itself was unknown.

## 5 CONCLUSION

To investigate the effect of long‐term continuous ECG monitoring on the assessment of HRV and HR dynamics, we analyzed the intraweek variations in HRV and HR dynamics indices during 7‐day continuous ECG monitoring in 107 outpatients with sinus rhythm. We found that (1) the intraweek coefficients of variance of HRV and HR dynamics indices ranged from 2.9 to 26.0%, and were smaller for frequency‐domain indices than for time‐domain indices and for indices reflecting slower HR fluctuations, (2) the indices with large variance, such as DC, SDNN, and VLF, often showed transient abnormalities from day to day over 7 days, reducing the positive predictive accuracy of the 24‐h ECG monitoring for detecting type 1 abnormalities, (3) 7‐day ECG monitoring provided 2.3‐ to 6.5‐fold increase in sensitivity to detect type 2 abnormalities of HRV and HR dynamics compared to 24‐h ECG monitoring, and (4) 7‐day ECG monitoring detected an average of 1.74 to 2.91 times as many abnormal values as 24‐h ECG monitoring. We conclude that long‐term continuous ECG increases the accuracy and sensitivity of detecting persistent and transient abnormalities in HRV and HR dynamics, and allows discrimination between the two types of abnormalities. In future studies, it would be desirable to investigate whether the identification of the two types of abnormalities by long‐term ECG can lead to improved risk stratification by HRV and HR dynamics.

## CONFLICT OF INTERESTS

Heart Beat Science Lab, Co. Ltd. has an advisory agreement with JSR Corporation.

## AUTHOR CONTRIBUTIONS

E.Y. and J.H. conceptualized the study; J.H. contributed to methodology and software; E.Y. and J.H. validated the study; E.Y. involved formal analysis; E.Y. investigated the study; J.H. and E.Y. contributed to resources; J.H. curated the data; J.H. wrote, reviewed, and edited; J.H. visualized the study; J.H. contributed to project administration; J.H. and Y.E. involved in funding acquisition. All authors have read and agreed to the published version of the manuscript.

## ETHICS

The protocol of study was approved by the Ethics Review Committee of the Nagoya City University Graduate School of Medical Sciences, Nagoya, Japan (No. 60–18–0211). Based on the protocol, informed consent for the research use of the anonymized ECG data was obtained from each subject by JSR Corporation (Japan).

## Data Availability

The data that support the findings of this study are available from JSR Corporation, Japan. Restrictions apply to the availability of these data, which were used under license for this study. Data are available from the authors with the permission of JSR Corporation, Japan.
